# Algal photophysiology drives darkening and melt of the Greenland Ice Sheet

**DOI:** 10.1073/pnas.1918412117

**Published:** 2020-02-24

**Authors:** Christopher J. Williamson, Joseph Cook, Andrew Tedstone, Marian Yallop, Jenine McCutcheon, Ewa Poniecka, Douglas Campbell, Tristram Irvine-Fynn, James McQuaid, Martyn Tranter, Rupert Perkins, Alexandre Anesio

**Affiliations:** ^a^Bristol Glaciology Centre, University of Bristol, Bristol BS8 1HH, United Kingdom;; ^b^Department of Geography and Earth Science, Aberystwyth University, Penglais, Aberystwyth, Ceredigion SY23 3FL, United Kingdom;; ^c^School of Biological Sciences, University of Bristol, Bristol BS8 1TQ, United Kingdom;; ^d^School of Earth and Environment, University of Leeds, Leeds LS2 9JT, United Kingdom;; ^e^School of Earth and Ocean Sciences, Cardiff University, Cardiff CF10 3AT, United Kingdom;; ^f^Department of Biology, Mount Allison University, Sackville, NB E4L3M7, Canada;; ^g^Department of Environmental Science, Aarhus University, 4000 Roskilde, Denmark

**Keywords:** Greenland Ice Sheet, glacier algae, photophysiology, melt, cryosphere

## Abstract

Blooms of Zygnematophycean “glacier algae” lower the bare ice albedo of the Greenland Ice Sheet (GrIS), amplifying summer energy absorption at the ice surface and enhancing meltwater runoff from the largest cryospheric contributor to contemporary sea-level rise. Here, we provide a step change in current understanding of algal-driven ice sheet darkening through quantification of the photophysiological mechanisms that allow glacier algae to thrive on and darken the bare ice surface. Significant secondary phenolic pigmentation (11 times the cellular content of chlorophyll *a*) enables glacier algae to tolerate extreme irradiance (up to ∼4,000 µmol photons⋅m^−2^⋅s^−1^) while simultaneously repurposing captured ultraviolet and short-wave radiation for melt generation. Total cellular energy absorption is increased 50-fold by phenolic pigmentation, while glacier algal chloroplasts positioned beneath shading pigments remain low-light–adapted (*E*_*k*_ ∼46 µmol photons⋅m^−2^⋅s^−1^) and dependent upon typical nonphotochemical quenching mechanisms for photoregulation. On the GrIS, glacier algae direct only ∼1 to 2.4% of incident energy to photochemistry versus 48 to 65% to ice surface melting, contributing an additional ∼1.86 cm water equivalent surface melt per day in patches of high algal abundance (∼10^4^ cells⋅mL^−1^). At the regional scale, surface darkening is driven by the direct and indirect impacts of glacier algae on ice albedo, with a significant negative relationship between broadband albedo (Moderate Resolution Imaging Spectroradiometer [MODIS]) and glacier algal biomass (*R*^2^ = 0.75, *n* = 149), indicating that up to 75% of the variability in albedo across the southwestern GrIS may be attributable to the presence of glacier algae.

## Significance

Processes that darken the surface of the Greenland Ice Sheet (GrIS) enhance energy absorption and accelerate melt, with consequences for global sea-level rise. Here, we demonstrate how summer blooms of “glacier algae” darken the ice surface, significantly impacting the physical integrity of the environment. We identify and quantify the energy regulation mechanisms employed by glacier algae to balance their requirements for photosynthesis and growth with the extreme light and temperature regime of the GrIS, demonstrating how these mechanisms are optimized to darken and melt the ice surface. Our findings are critical for the incorporation of biological feedbacks into predictive models of GrIS surface runoff and provide unique insight into how photoautotrophic life excels within icy environments.

Melting of the Greenland Ice Sheet (GrIS), the second-largest body of ice in the world, is the single largest cryospheric contributor to global eustatic sea-level rise (1). From 1991 to 2011, a mass loss of 2.9 ± 0.5 × 10^3^ Gt of ice resulted in an equivalent 8-mm mean global sea-level rise, with the GrIS currently contributing ∼2 mm⋅y^−1^ (2). Increasing GrIS mass loss is dominated by surface melt (61%) as opposed to solid ice discharge, which in turn is controlled by surface albedo (2–4). As albedo declines, darker ice absorbs increasing amounts of shortwave radiation, enhancing melt. Accordingly, long-term declines in GrIS surface albedo have paralleled accelerated surface melt (2, 3, 5), particularly along the western margin of the ice sheet in the so-called dark-zone (6, 7). Processes that serve to darken the GrIS surface thus hold significant potential to impact melt, with global consequences.

Deposition and/or melt-out of mineral dust, soots from incomplete combustion from anthropogenic sources (termed “black carbon”) or forest fires (“brown carbon”), and the accumulation of pigmented photoautotrophs (agents of “biological albedo decline”) all represent light-absorbing impurities that darken ice surfaces (7). Of these, biologically driven albedo reduction has been proposed by both observational (7–10), and modeling studies (11) to represent the single largest contributor to albedo decline in the GrIS dark zone in recent decades, matching reports from other regions of the cryosphere (12–14). Supraglacial photoautotrophic populations of the GrIS include cyanobacteria, typically associated with aggregates of inorganic particles (cryoconite) that melt down into the ice to form water-filled depressions (cryoconite holes) (15–18), and heavily pigmented Zygnematophycean (Streptophyte) microalgae (hereafter glacier algae) (19) that bloom in the upper few centimeters of surface ice, which is subsequently described as dark or dirty ice (8, 9, 19–23). Given the high abundance and large spatial coverage achieved by blooms of glacier algae during summer ablation seasons (8, 9, 23), glacier algal assemblages represent the most important photoautotrophic component of the GrIS supraglacial environment with regard to biological albedo effects (8, 9, 11).

The supraglacial surface on which glacier algal blooms occur is characterized by extremes in environmental stressors. Amplified seasonal patterns in irradiance, temperature, and water availability necessitate survival for months in total darkness at subzero conditions, followed by short (∼3 mo) summer ablation periods characterized by photoinhibitory levels of irradiance, high ultraviolet (UV) radiation, and diurnal freeze–thaw cycles (8, 18, 19, 24, 25). Photoautotrophs, which represent the essential base of inorganic carbon fixation and autotrophic energy production in cold ecosystems, must balance their light-harvesting requirements for photosynthesis and the potential thermal benefits of localized warming conferred by energy capture with the detrimental effects of overexcitation of the photosynthetic apparatus and excess UV exposure. For glacier algae photosynthesizing in supraglacial environments, the production of a unique purpurogallin phenolic pigment, purpurogallin carboxylic acid-6-*O*-β-d-glucopyranosidel (26), in addition to the suite of light harvesting and photoprotective pigments typical of green microalgae (23, 26, 27), has been postulated to provide photoprotection against excessive UV and visible irradiance (23, 26, 27) and to potentially serve as a mechanism to generate heat and thus liquid water surrounding the cell (28). This pigmentation also likely represents the major agent of biological albedo decline and enhanced surface melt associated with glacier algal blooms (8, 9, 11, 28).

Despite the global importance of glacier algal-driven pigment accumulation within GrIS surface ice, the link between glacier algal photoprotection, cellular heat generation, and pigment regulation has as yet only been postulated. To date, no study has analyzed the photophysiological mechanisms employed by glacier algae to regulate their photosynthetic apparatus relative to the light environment of the GrIS, or the quantitative potential of secondary pigmentation for energy capture and melt generation. Furthermore, previous studies have not assessed potential dynamism and limitations of these mechanisms and the net outcomes for pigment accumulation and surface darkening through the biological albedo effect. Here, we assess the mechanisms driving biological darkening of the GrIS. We determined the photoacclimation and regulation mechanisms employed by glacier algae to balance excitation pressure within photosystem II (PSII), the roles and relative importance of purpurogallin versus typical photoprotective pigments in these processes, the net outcomes for energy capture and utilization by glacier algal cells, and the consequences for biological albedo decline across an entire melt season in the southwestern GrIS.

**Fig. 1.**
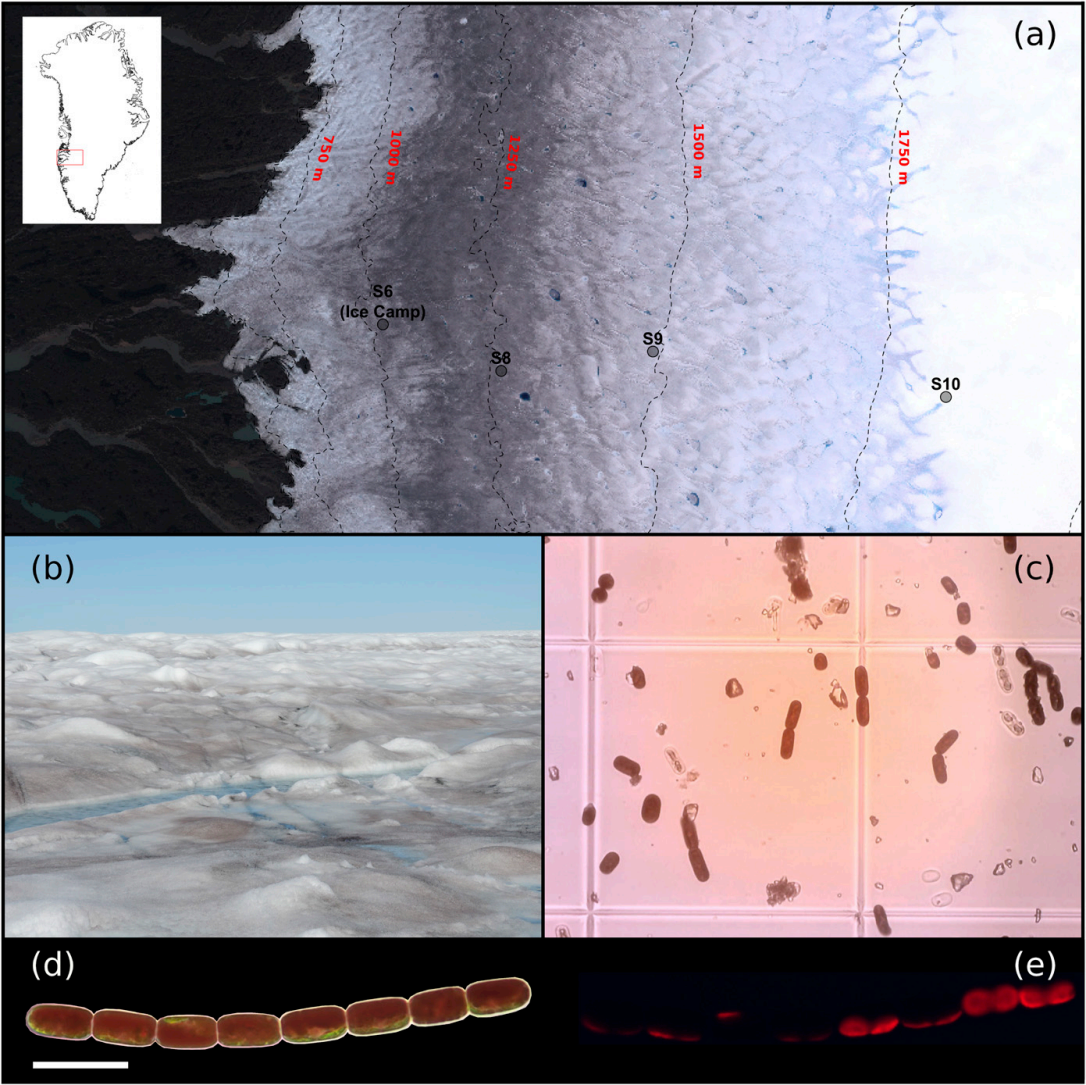
Glacier algae and the surface of the GrIS. (*A*) The southwestern GrIS margin near Kangerlussuaq, showing sampling (S6, primary ice camp) and modeling site locations (sites S6 through S10) across the K-transect. Note the conspicuous “dark zone” running parallel to the ice sheet margin for which glacier algal blooms are thought responsible. (*B*) GrIS surface ice at our primary ice camp location dominated by a glacier algal bloom during the 2016 ablation season. (*C*) Heavily pigmented glacier algal assemblages sampled from the surface of the GrIS. (*D*) *A. nordenskiöldii* filament showing pancake-shaped chloroplasts located beneath abundant secondary phenolic pigmentation. (*E*) Chloroplast location within the cells highlighted by epifluorescence microscopy. (Scale bars in *D* and *E*, 50 µm.)

All research was performed in situ on the southwestern GrIS, ∼35 km inland of the western ice margin (Fig. 1), at a primary ice camp established throughout the 2016 ablation season (early July to late August). A well-developed bloom of Zygnematophycean glacier algae was present in surface ice throughout the ablation period, dominated by two ice environment specialist taxa, *Ancylonema nordenskiöldii* and *Mesotaenium berggrenii* (see ref. 23 for a description of general bloom dynamics). Here, the photophysiology of supraglacial glacier algal communities was assessed using pulse-amplitude modulation (PAM) fluorometry twinned with high-performance liquid chromatography (HPLC) profiling of all algal pigments. Key light use mechanisms were determined as well as the response of glacier algal communities to in situ irradiance regimes. The role of phenolic shading pigments in glacier algal photoprotection was demonstrated by quantifying the spectral mass absorption coefficients of glacier algal phenolic extracts and recalculating the incident excitation captured by glacier algal chloroplasts with/without their presence. In parallel, glacier algal dependence on typical nonphotochemical quenching (NPQ) mechanisms for photoprotection was investigated using inhibitor incubations. Taken together, data allowed estimation of the energy budget for a glacier algal cell, demonstrating the distribution of captured energy between photochemistry and melt generation. Finally, the net outcomes of glacier algae photophysiology and pigment regulation for darkening of the GrIS were demonstrated by monitoring and modeling pigment profiles and biomass accumulation within surface ice over the 2016 ablation season, with subsequent modeling of impacts to surface ice albedo using the BioSNICAR-GO radiative transfer model of ref. 11 and comparisons to MODIS (Moderate Resolution Imaging Spectroradiometer) satellite-derived broadband albedo (BBA) measurements. These results significantly advance our current understanding of the biological mechanisms that underpin ice sheet surface mass balance and seasonal runoff generation.

## Results and Discussion

### High-Light Acclimation of Glacier Algal Assemblages.

**Fig. 2.**
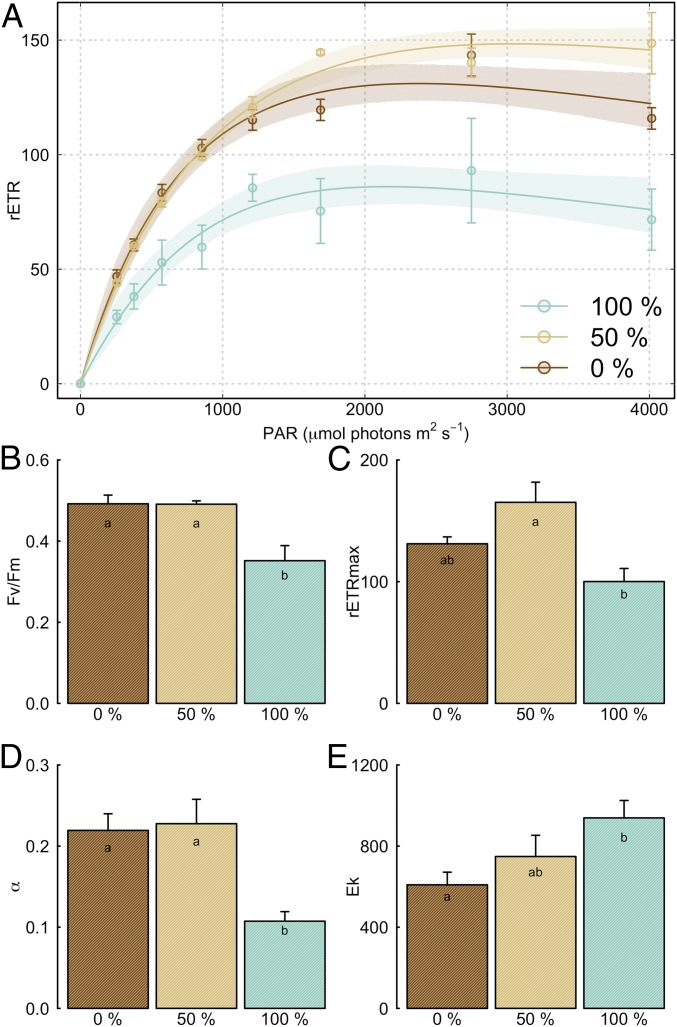
Whole-cell glacier algae photophysiology following 24-h incubation under 0% (dark brown), 50% (light brown) and 100% (light green) ambient irradiance on the GrIS surface, showing rapid light response curves (RLCs, *A*) and derived parameters (*B*–*E*). (*A*) Relative electron transport rates (rETR) measured during RLCs (circles), with modeled rETR (46) (solid lines) and 95% confidence intervals (shaded regions). (*B*) Maximum quantum efficiency in the dark-adapted state (*F*_*v*_*/F*_*m*_). (*C*) Maximum rate of relative electron transport (*rETR*_*max*_). (*D*) Light utilization efficiency (*α*). (*E*) Light saturation coefficient (*E*_*k*_). All panels show mean ± SE, *n* = 3. Lowercase letters in *B*–*E* denote homogenous subsets determined from one-way ANOVA analysis of respective parameters in relation to irradiance treatment (*F*_*2,8*_ = 10.23, 7.58, 9.24, and 3.69, respectively, *P* < 0.05 in all cases).

The photophysiology of glacier algal communities sampled from the surface of the GrIS was assessed following 24-h incubation under 100%, 50%, and 0% ambient irradiance (*Methods*), revealing their capacity to tolerate extreme levels of irradiance (Fig. 2). While photoacclimation to high-light environments has previously been postulated for glacier algal taxa (e.g., refs. 8, 23, 26, and 27), it has not been constrained until now, with the single previous attempt at fluorescence-based assessment of GrIS supraglacial glacier algal communities (8) unable to produce saturating light curves given the lower range of photosynthetically active radiation (PAR) employed. By pushing rapid light curves (RLCs; ref. 29) to extreme levels of incident irradiance (∼4,000 µmol photons⋅m^−2^⋅s^−1^), we were able to force glacier algal PSII reaction centers to saturation, enabling determination of key features of their photophysiology. We do not contend that in situ reaction centers were naturally receiving the incident excitation applied during RLCs (discussed below), but rather these data demonstrate the net outcomes of glacier algae optimal photophysiology for energy regulation and tolerance (Fig. 2).

The onset of saturation of electron transport through PSII (*E*_*k*_) averaged 938 ± 149 µmol photons⋅m^−2^⋅s^−1^ after 24-h incubation under 100% ambient irradiance, with total saturation apparent at ∼2,000 µmol photons⋅m^−2^⋅s^−1^ across treatments, and sustained electron transport for up to 20 s at 4,000 µmol photons⋅m^−2^⋅s^−1^ incident irradiance (Fig. 2*A*). This highlights the significant capacity of glacier algal cells to tolerate extreme incident irradiance. Across incubations, suppression of glacier algal photochemistry by high-light-induced photoinhibition was also apparent, with maximum quantum efficiencies (*F*_*v*_*/F*_*m*_, an inverse proxy of stress in microalgae), maximum rates of electron transport (*rETR*_*max*_, a proxy for the rate of photosynthesis), and light utilization efficiencies (*α*) all significantly lower under 100% as compared to 50% or 0% ambient irradiance after 24 h (Fig. 2 *B*–*E*). While glacier algal assemblages were able to transiently tolerate irradiance up to 4,000 µmol photons⋅m^−2^⋅s^−1^, the irradiance apparent on the GrIS during the midablation season (∼1,700 µmol photons⋅m^−2^⋅s^−1^) was sufficient to suppress glacier algae photochemistry, representing a potential limitation on productivity and growth.

Given that physiological mechanisms including shading pigments can serve to lower the irradiance actually received by microalgal chloroplasts (30, 31), orientation of glacier algal chloroplasts beneath vacuoles filled with phenolic pigmentation (Fig. 1 and refs. 23, 26, 27, 32, and 33) likely served to intercept a significant portion of the incident irradiance applied during RLCs, causing overestimation of electron transport rates and *E*_*k*_. By dissipating the intercepted incident irradiance as heat, this secondary pigmentation may also serve to generate liquid water surrounding the cells (28). To both constrain the actual photosynthetic responses of glacier algal chloroplasts and to estimate the magnitude of algal energy capture directed to ice surface melting, the light attenuation provided by phenolic pigmentation must be assessed.

### Secondary Phenolic Pigmentation Dominates Light Absorption.

To determine the cellular content and biooptical properties of glacier algal phenolic pigmentation, the complete suite of glacier algal pigments (chlorophylls, carotenoids, and phenols) were extracted and quantified using a combination of HPLC and spectrophotometric assays (*Methods*). Mass-specific absorption coefficients (m^2^⋅mg^−1^⋅nm^−1^, λ_250–750nm_) were derived for glacier algal phenols by quantifying their concentration in GrIS surface ice samples containing a range of glacier algal abundance (187 cells⋅mL^−1^ to 2.1 × 10^4^ cells⋅mL^−1^, *n* = 53), with parallel assessment of the spectral light absorption of extracts provided by spectrophotometric wavescans (*Methods*). Single-cell absorption cross-sections (m^2^⋅cell^−1^) were then reconstructed using the in vivo mass-specific absorption coefficients of the major pigment classes ($a_{i\left(\lambda \right)}$, m^2^⋅mg^−1^: phenolics derived here; with values for chlorophyll *a*, chlorophyll *b*, photosynthetic carotenoids, and photoprotective carotenoids from ref. 34) multiplied by their cellular content ($C_{i}$, mg⋅cell^−1^), as $a_{\left(\lambda \right)}=\sum a_{i\left(\lambda \right)}\times C_{i}$. This approach uses specific absorption coefficients accounting for wavelength shifts produced by protein binding of major chlorophyll and carotenoid pigments within algal cells but does not correct for potential pigment packaging effects caused by self-shading of pigments as the content per biovolume increases (35).

**Fig. 3.**
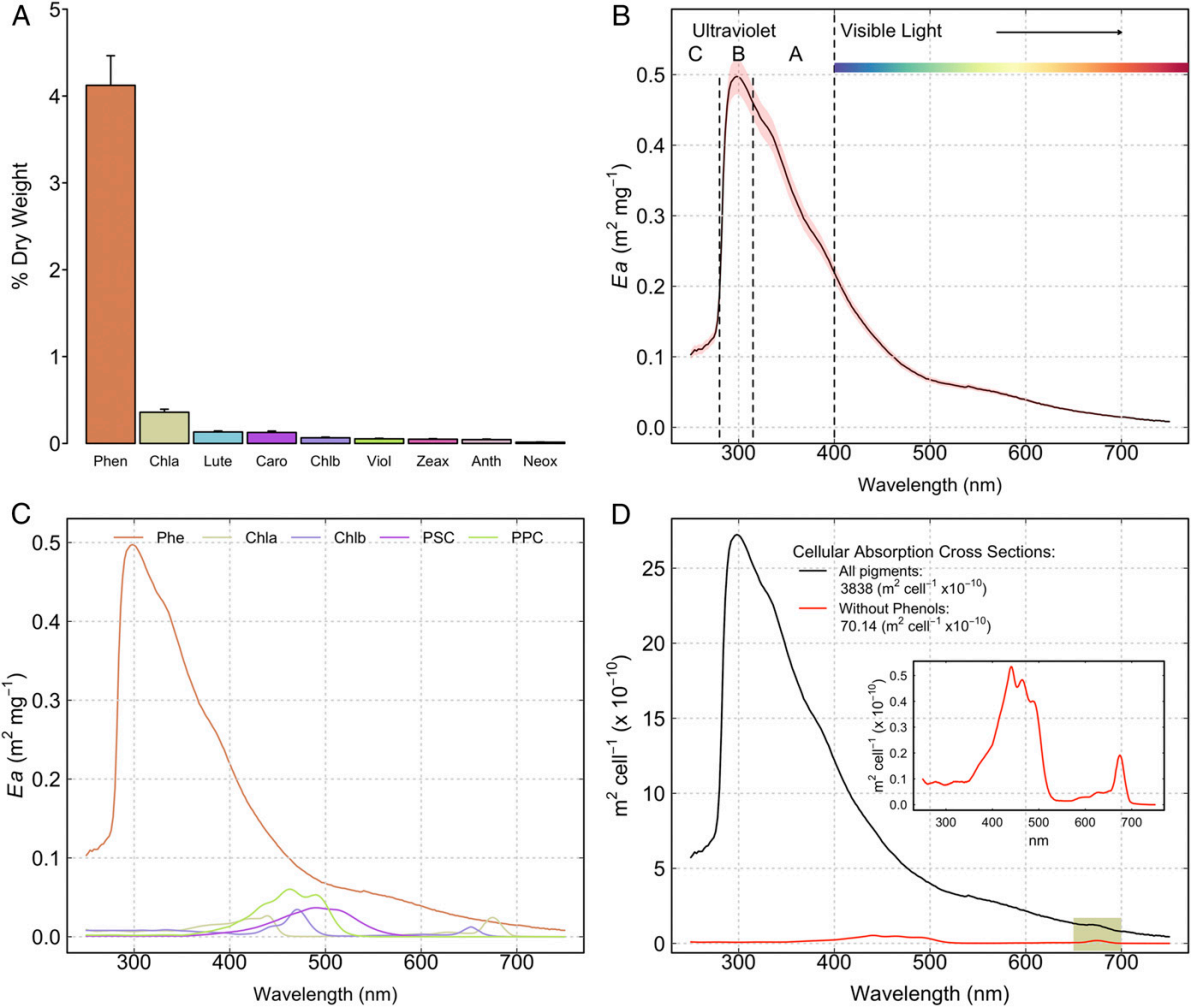
Glacier algal pigmentation and biooptical properties. (*A*) Cellular contents of all glacier algal pigments, including phenolics (Phen), chlorophyll *a* (Chl*a*), lutein (Lut), β-carotene (Caro), chlorophyll *b* (Chlb), violaxanthin (Viol), zeaxanthin (Zea), antheraxanthin (Anth), and neoxanthin (Neox). (*B*) Mass absorption coefficients of glacier algal phenolic extracts across the UV and visible spectrum. (*C*) Comparison of the mass absorption coefficients of all major pigment types (phenolics [Phe] measured here and chlorophyll *a* [Chl*a*], chlorophyll *b* [Chl*b*], photosynthetic carotenoids [PSC], and photoprotective carotenoids [PPC] from ref. 34). (*D*) Glacier algae single-cell absorption cross-sections, showing absorption cross-sections for the complete complement of glacier algal pigments (black line), absorbance without secondary phenolic pigmentation (red line and *Inset*), and highlighting the notable absorption feature related to chlorophyll *a* at λ_675nm_ (green shaded region).

Glacier algal cellular phenolic contents were significantly increased as compared to other light-harvesting and photoprotective pigments, with phenolic content ∼11 times the content of chlorophyll *a* (Fig. 3*A*). This shows a significant metabolic investment in photoprotective as opposed to light-harvesting pigmentation within glacier algal cells, consistent with our photophysiology data showing tolerance of high incident irradiance. Phenolic extracts demonstrated their greatest absorbance in the UV-B ($\lambda_{max}=300\;\mathrm{nm}$, Fig. 3*B*), with significant absorbance also across the UV-A (secondary shoulder at λ=335 nm), and a broad but decreasing absorbance across the visible spectrum to the red (Fig. 3*B*). HPLC assessment of phenolic extracts confirmed the presence of four major compounds, the first absorbing solely in the UV $\left(\lambda_{max}=278\;\mathrm{nm}\right)$, with the remainder showing identical absorbance features in both the UV and across the visible spectrum ($\lambda_{max}=304\;\mathrm{nm}$, secondary peak at λ=389 nm) (*SI Appendix*, Fig. S1), consistent with the only previous characterization of glacier algal phenolics (26). Mass-specific absorption coefficients of phenolics were high compared to those of other major pigment classes (Fig. 3*C* and refs. 34 and 35), which combined with their high cellular contents (Fig. 3*A*) gave an overwhelming influence on reconstructed glacier algal cellular absorption cross-sections (Fig. 3*D* and *Methods*).

Light absorption by glacier algal cells was increased ∼50-fold by their abundant phenolic pigmentation (Fig. 3*D*), with spectrally integrated cellular absorption cross-sections $\left(\lambda_{250-750\text{nm}}\right)$ increasing from ∼70 × 10^−10^ m^2^⋅cell^−1^ for cross-sections reconstructed without phenolics (Fig. 3 *D*, *Inset*) to 3,838 × 10^−10^ m^2^⋅cell^−1^ in their presence. Phenolics thus unequivocally constitute the major light absorber within glacier algal cells and are consequently the major mechanism underlying biological albedo decline associated with glacier algal blooms (9, 11). Given their decreasing absorbance across the visible spectrum, glacier algal phenolics particularly serve to absorb UV and high-energy blue visible radiation, while permitting preferential light harvesting at longer, less-damaging wavelengths (36); note the chlorophyll *a* absorption feature still evident at ∼$\lambda_{675\text{nm}}$ (Fig. 3*D*).

**Fig. 4.**
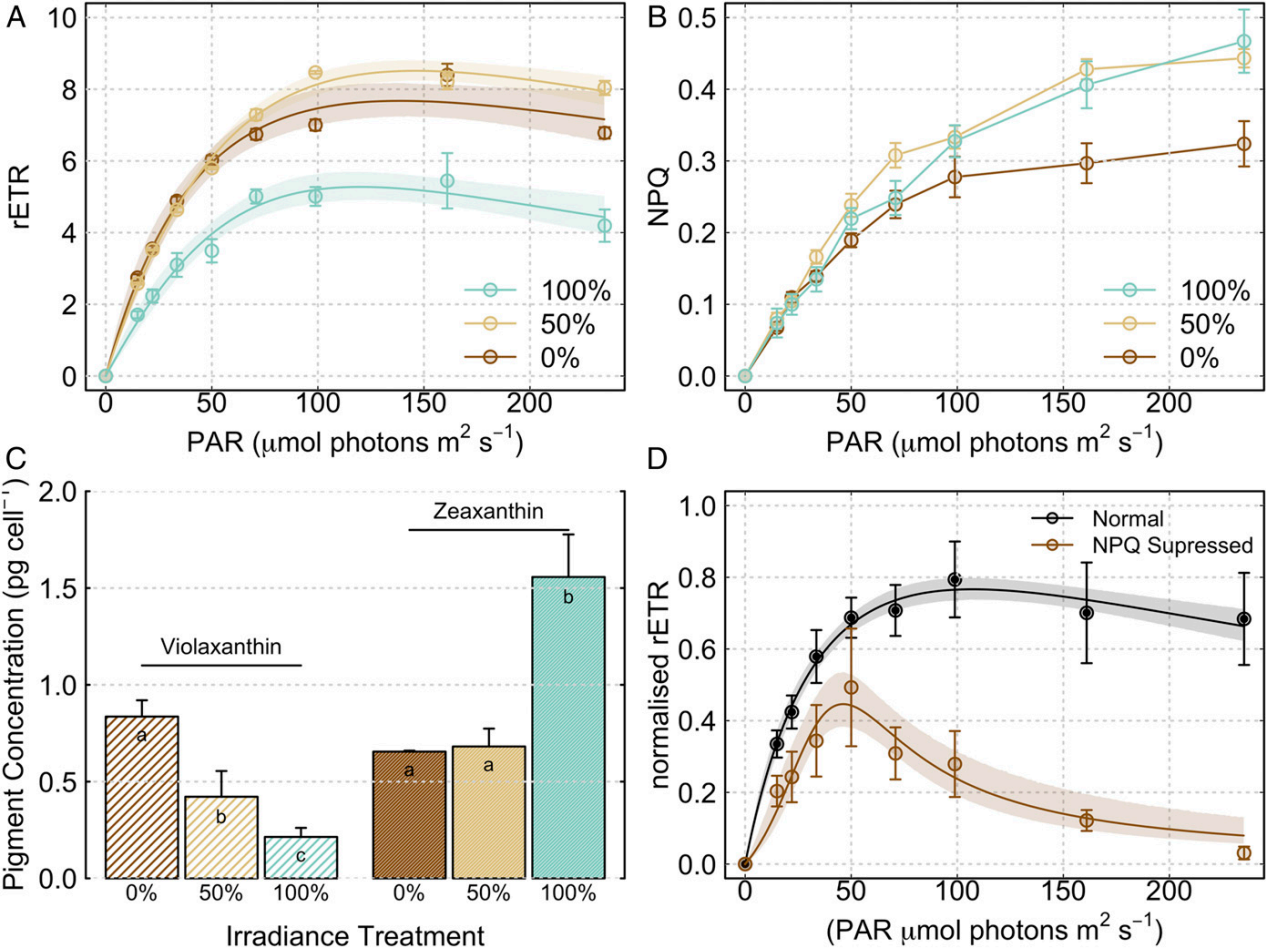
Chloroplast-level glacier algae photophysiology (i.e., corrected for shading by phenolic pigmentation). (*A*) Rapid light response curves (RLCs) following 24-h incubation under 0% (dark brown), 50% (light brown), or 100% (light green) ambient irradiance [points show measured relative electron transport rates (rETR), lines show modeled rETR after (46), and shaded regions show 95% confidence intervals]. (*B*) NPQ recorded throughout RLCs across light treatments. (*C*) Violaxanthin and zeaxanthin cellular pigment concentrations across light treatments at the cessation of incubations (lowercase letters denote homogeneous subsets identified from ANOVA analysis of respective pigment concentrations in relation to light treatment). (*D*) RLCs of control and NPQ-inhibited (i.e., DTT-treated) glacier algal assemblages. All plots show mean ± SE, *n* = 3.

During the present study, photophysiology was assessed using a WaterPAM fluorometer (Walz GmBH) with measuring and actinic irradiance centered around $\lambda_{660\text{nm}}$, at which glacier algal phenolics were responsible for ∼94% of the total cellular absorption cross-section. To correct for light attenuation provided by phenolic pigmentation and estimate the actual photosynthetic response of glacier algal chloroplasts, the incident excitation applied during RLCs was empirically reduced based on absorbance of phenolics at $\lambda_{660\text{nm}}$ and electron transport rates recalculated from PSII quantum efficiencies (*Methods*). This correction shifted the onset of light saturation for glacier algal chloroplasts (*E*_*k*__*-*__*chloroplast*_) to ∼46 ± 13 µmol photons⋅m^−2^⋅s^−1^ (Fig. 4*A*), significantly lower than the total cell *E*_*k*_ reported above (Fig. 2*E*), with a parallel reduction in the maximum rate of electron transport through PSII (*rETR*_*max*_). Glacier algal chloroplasts located beneath secondary phenolic pigmentation (Fig. 1) thus remain comparatively low-light–adapted despite the high-light environment of GrIS surface ice during summer ablation seasons, whereby PAR in excess of 1,600 µmol photons⋅m^−2^⋅s^−1^ is common during clear-sky days. These data confirm the dependence of glacier algal photosystems on shading by phenolics to limit photoinhibition of photosynthesis. This adaptation to preempt photoinhibition is particularly critical, since the counteracting repair is slow at low temperature (37).

### Reliance on Typical NPQ Mechanisms.

Additional to secondary phenolic pigmentation, glacier algal cells remained dependent on typical NPQ mechanisms for protection of their photosystems (Fig. 4 *A* and *B*). In green algae, reversible induction of NPQ is mediated primarily by the light-driven deepoxidation of specific xanthophyll pigments, violaxanthin to antheraxanthin, and finally to zeaxanthin, with recovery of initial pigment pools in the dark (38, 39). We found that NPQ increased with irradiance over RLCs across all incubations, with progressive saturation at PAR > *E*_*k*__-__chloroplast_ (∼46 µmol photons⋅m^−2^⋅s^−1^). In parallel, decreases in violaxanthin (initial xanthophyll-cycle pigment pool) and increases in zeaxanthin (terminal xanthophyll-cycle pigment pool) indicated progressive dependence on xanthophyll-cycle-driven NPQ with increasing irradiance across treatments (Fig. 4*C*). In contrast, no change was apparent in the cellular content of phenolics after 24 h incubation (0.041 ± 0.001 ng phenol⋅cell^−1^). Glacier algal phenolic pigmentation therefore does not represent a rapidly inducible form of photoregulation but rather a sustained screening capacity. To verify dependence on NPQ, RLCs were performed on field-collected glacier algal assemblages ± an inhibitor of NPQ (Fig. 4*D* and *Methods*). At all light levels (corrected for shading of the chloroplasts by phenolic pigmentation at measurement wavelength), samples with chemically suppressed NPQ demonstrated lower quantum yields of PSII and corresponding decreased electron transport rates relative to control samples. Above ∼50 µmol photons⋅m^−2^⋅s^−1^, glacier algae with inhibited NPQ demonstrated a rapid decline in electron transport, with electron transport rates approaching zero by ∼250 µmol photons⋅m^−2^⋅s^−1^. Given that photoinhibition was apparent under 100% ambient irradiance (Fig. 4*A*), NPQ was not sufficient to fully protect glacier algal photosystems in situ. Increased photoinhibition and reduced capacity for photosynthesis would therefore be predicted during midablation periods when 24-h irradiance prevails at higher latitudes.

### Cellular Energy Budget and Radiative Forcing.

**Fig. 5.**
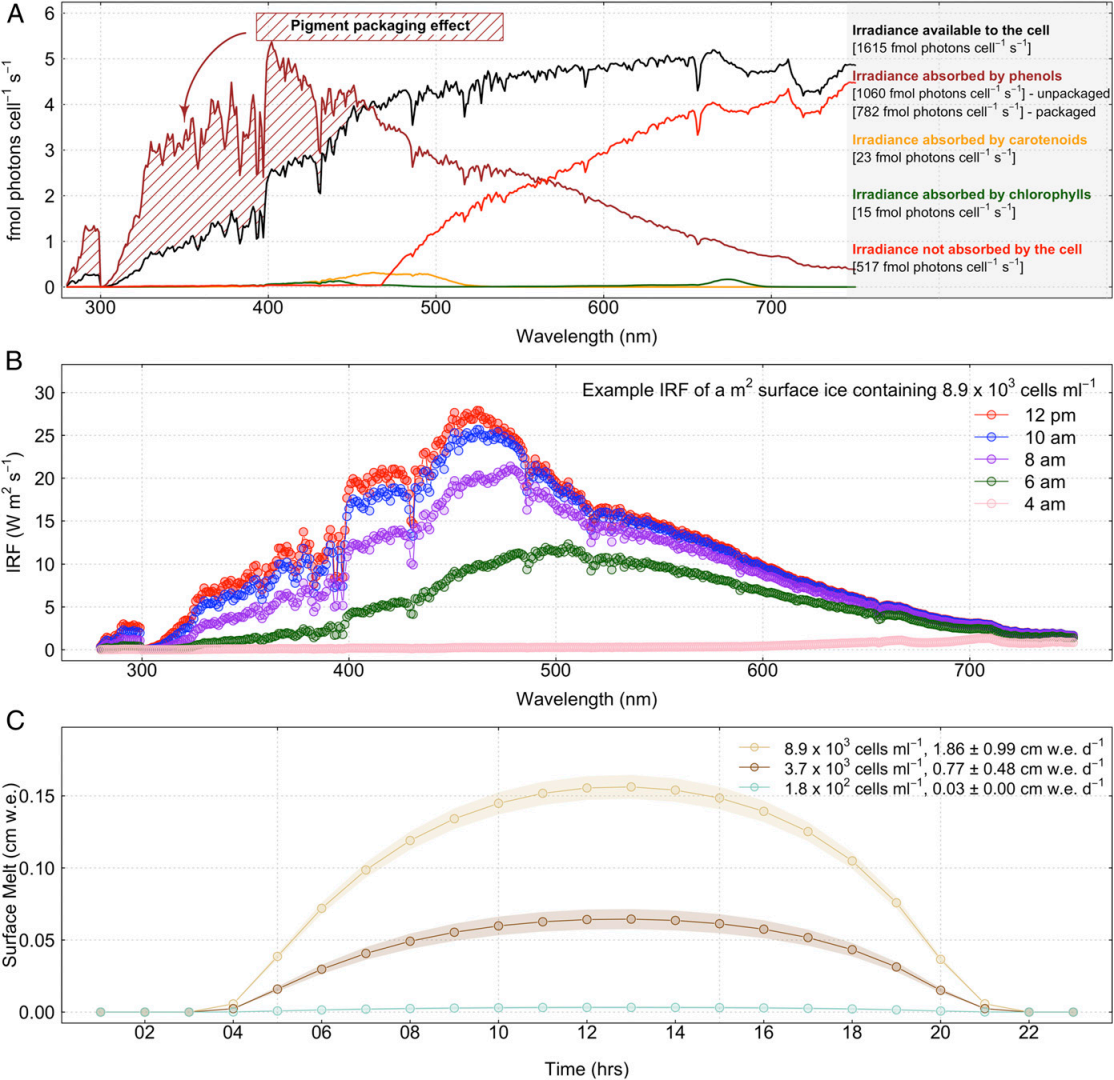
Glacier algae cellular light capture, IRF, and melt generation potential. (*A*) Glacier algal spectral irradiance absorption (fmol photons⋅cell^−1^⋅s^−1^) portioned into the major pigment classes (phenolics, carotenoids, and chlorophylls) in relation to total incident irradiance available to the cell. (*B*) The IRF (W⋅m^−2^⋅s^−1^) of a square meter of surface ice containing a high abundance (8.9 × 10^3^ cells⋅mL^−1^) of glacier algal cells throughout the diel cycle. (*C*) Mean additional hourly surface melt generation (cm w.e.) estimated for 1 m^2^ of surface ice containing a low (186 ± 276 cells⋅m^−1^, *n* = 27, light green data), medium (3,711 ± 2,333 cells⋅m^−1^, *n* = 34, dark brown data), or high (8,989 ± 4,773 cells⋅m^−1^, *n* = 103, light brown data) abundance of glacier algae on 26 July 2016 at our primary ice camp location. Plot shows mean meltwater generation per abundance category (points) ± SE (shaded regions).

Collectively, our data allow estimation of the light energy budget for a glacier algal cell, providing estimation of the energy utilized for photochemistry versus that available for ice surface melting (Fig. 5). Midday (12 PM) spectral irradiance was obtained for our ice camp location at 1-nm resolution using the PVSystems solar irradiance program for 26 July 2016 following ref. 28 and the amount of light energy absorbed by a representative glacier algal cell (fmol⋅photons⋅s^−1^⋅nm^−1^) calculated by multiplying total cellular absorption cross-sections (Fig. 3, expressed in m^2^⋅cell^−1^⋅nm^−1^) by incoming spectral irradiance (expressed as μmol photons⋅m^−2^⋅s^−1^⋅nm^−1^). The same approach, which assumes 100% down-welling irradiance, was further applied to calculate the proportion of irradiance absorbed by different glacier algal pigment components (*Methods*).

Glacier algal cells absorbed all incident light below ∼465 nm, consistent with the increasing contribution of phenolics to total cell absorption as wavelength decreases (Fig. 5*A*). Predicted irradiance capture exceeded available irradiance at lower wavelengths (Fig. 5*A*, brown shaded area), likely reflecting pigment packaging effects within the cell not accounted for in the mass-specific absorption cross-sections. As pigment concentrations increase within algal cells, the effective absorption per unit pigment decreases due to self-shading (35). Correcting for this packaging effect (*Methods*) demonstrated that glacier algal phenolics capture ∼48% of the irradiance incident upon the cell, with minor contributions to absorbance from total carotenoids (1.4%) and chlorophylls (0.93%). Light capture for photochemistry thus equated to just ∼1 to 2.4% of the total irradiance incident on the cell, with ∼32% of available irradiance not absorbed, and the remainder potentially available for melt generation.

To estimate the additional melt generation driven by a low (186 ± 276 cells⋅mL^−1^), medium (3,711 ± 2,333 cells⋅mL^−1^), or high (8,989 ± 4,773 cells⋅mL^−1^) abundance of glacier algal cells within 1 m^2^ of surface ice (categories reflecting ref. 23), the hourly instantaneous radiative forcing (IRF) was approximated as above using hourly estimates of instantaneous spectral irradiance over the complete diel cycle (expressed as W⋅m^−2^⋅nm^−1^ and multiplied by 3,600 s⋅h^−1^), with correction for pigment-packaging effects (*Methods*). Specific meltwater equivalent (as cm w.e.) was derived by converting square meters to square centimeters and dividing by the latent heat of fusion for melting ice (334 J⋅cm^3^) (Fig. 5 *B* and *C*). The IRF progressed throughout the diel cycle concomitant with incoming spectral irradiance (Fig. 5*B*), peaking at solar noon with an hourly melt generation potential of 0.003 ± 0.004, 0.062 ± 0.039, and 0.155 ± 0.082 cm w.e. for ice containing a low, medium, or high glacier algal abundance, respectively (Fig. 5*C*). Integration over the complete diel cycle revealed the potential for glacier algal assemblages to contribute from 0.03 ± 0.00 cm w.e.⋅d^−1^ in low-biomass areas (mean ± SE, *n* = 27) up to 1.86 ± 0.99 cm w.e.⋅d^−1^ melt production in high-biomass patches of surface ice (mean ± SE, *n* = 103) (Fig. 5*C*), consistent with estimates derived by spectral differencing between sites containing glacier algae with those of “clean ice” at our primary ice camp location (1.35 ± 0.01 cm w.e.; ref. 11).

### Consequences for Darkening of the GrIS.

To understand the significance of glacier algae energy capture for GrIS surface darkening at the regional scale, pigment profiles of glacier algal assemblages were determined at regular intervals over the 2016 ablation season and combined with information on spatially averaged biomass loadings within surface ice estimated using a reanalysis of ref. 23 glacier algal bloom development model, forced here by shortwave-down radiation and temperature (*Methods* and *SI Appendix*). This represents the first model of glacier algal bloom development on the surface of the GrIS driven by physical parameters. Estimated algal biomass and measured pigment profiles were then used to drive the two-stream BioSNICAR-GO radiative transfer model of ref. 11 (*Methods*) to calculate ice surface BBA over the visible spectrum (λ_350–700nm_), across which glacier algal pigments absorb (Fig. 3). Verification of outputs was achieved by comparison of glacier algal biomass with BBA estimates using our radiative transfer approach and measurements of BBA (λ_400–3,000nm_, ref. 40) derived from MODIS satellite observations over the study period. To highlight the larger-scale consequences of glacier algal bloom development on GrIS surface darkening, we applied our model to several locations along the well-described K-transect that spans the southwestern GrIS ablation zone (Fig. 1), across which we have focused recent research (e.g., refs. 7, 11, 22, 23, and 41).

**Fig. 6.**
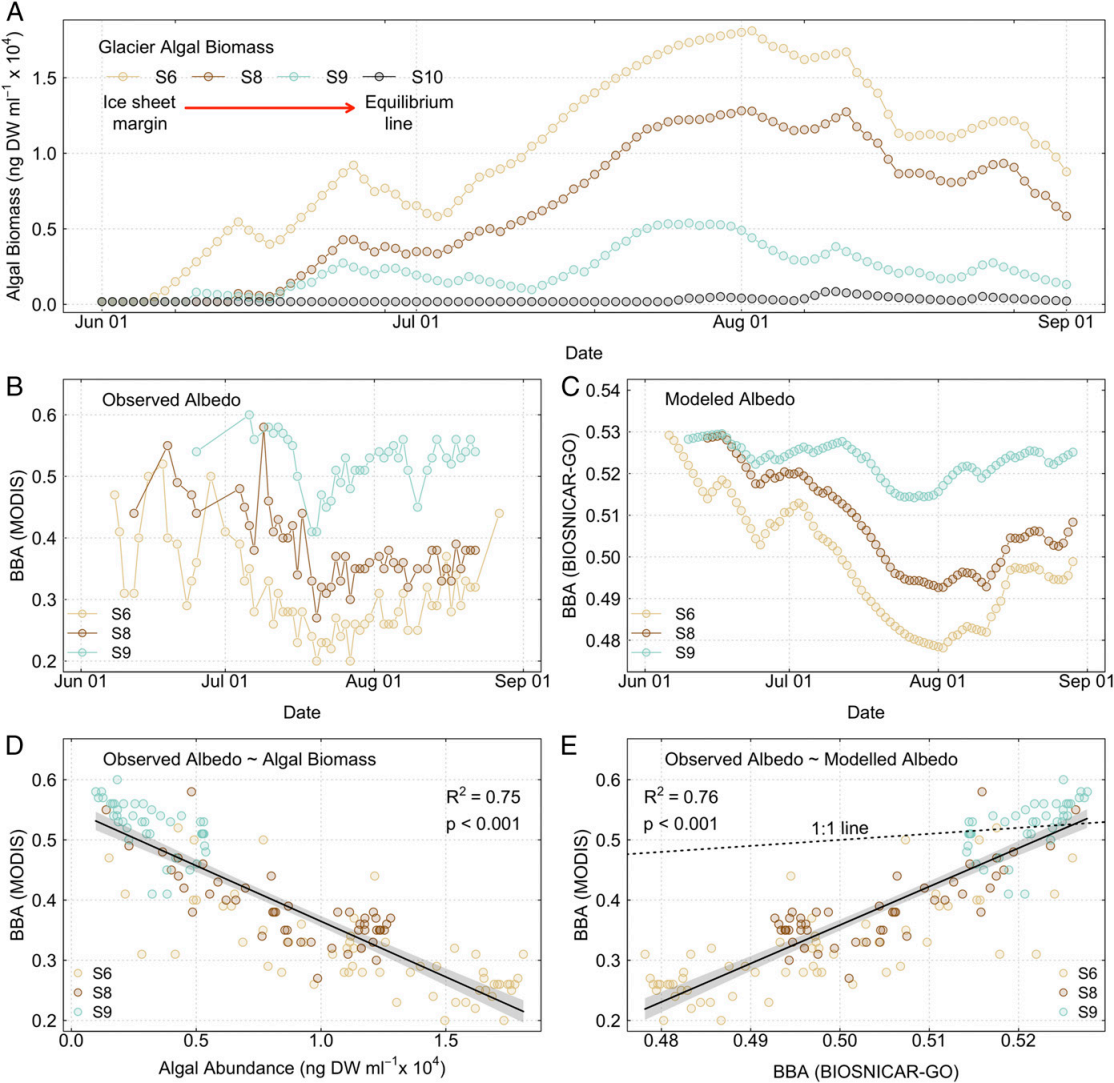
Glacier algae biomass accumulation in GrIS surface ice and relationships to surface darkening. (*A*) Modeled glacier algae biomass within surface ice across the southwestern GrIS (K-transect) throughout the 2016 ablation period. (*B*) Surface ice BBA measured by MODIS satellite observations across the K-transect over our study period. (*C*) Modeled (BioSNICAR-GO) surface ice visible BBA (λ_350–750nm_) across the same sites and time period. (*D*) Least squares linear regression of observed BBA (MODIS) in relation to modeled glacier algal biomass across sites, showing regression fit (black line), 95% confidence intervals (gray shaded area), coefficient of determination (*R*^*2*^), and associated significance (*P* value). (*E*) Least squares linear regression of measured BBA (MODIS) in relation to modeled BBA (BioSNICAR-GO), showing regression fit (black line), 95% confidence intervals (gray shaded area), coefficient of determination (*R*^*2*^), associated significance (*P* value), and 1:1 relationship (black dotted line).

Modeled glacier algal biomass (Fig. 6*A*) showed spatiotemporal patterning highly consistent with observed bloom development during the 2016 ablation season (23), with maximal biomass accumulation apparent at the most marginal site [S6, 1,075 m above sea level (a.s.l.), carrying capacity = 14,902 ± 500 ng dry weight (DW)⋅mL^−1^], and the lowest biomass apparent in surface ice at S10 located above the equilibrium line in 2016 (1,877 m a.s.l., carrying capacity = 449 ± 29 ng DW⋅mL^−1^). Longer bare-ice melt duration thus promoted algal biomass development, likely through enhanced meltwater and nutrient availability, solar radiation input, and diminished snow cover (19). Biomass accumulation peaked within surface ice from mid-July to early August 2016 across S6, S8, and S9, corresponding to the lowest ice surface albedo measured by MODIS (Fig. 6*B*) and modeled using our radiative transfer approach (Fig. 6*C*). Thereafter, decreases in biomass were predicted across all sites until the end of the ablation season concomitant with increases in surface ice albedo, with final concentrations of glacier algae in surface ice ranging from 309 ng DW⋅mL^−1^ at S10 to ∼1.0 × 10^4^ ng DW⋅mL^−1^ at S6. While the fate of glacier algal biomass remaining in surface ice over the winter period is currently unknown (19), some retention until the proceeding ablation season is expected given long-term decreases in GrIS surface albedo (2, 3) that may indicate interannual accumulation of autochthonous organic matter (7) and observation of active glacier algal communities at our study site prior to snow line retreat in 2017 (41).

Glacier algal biomass showed a significant negative linear relationship with surface ice BBA derived from MODIS satellite observations (*R*^2^ = 0.75, *P* < 0.001, *n* = 149; Fig. 6*D*), indicating that up to 75% of the variability in albedo across our southwesterly GrIS transect may be attributable to the presence of glacier algae. Disparity between the magnitude of albedo reduction modeled using BioSNICAR-GO across the visible spectrum (λ_350–700nm_, Fig. 6*C*) and those measured by satellite observations extending into the short-wave infrared wavelengths (λ_400–3,000nm_, Fig. 6*B*) further highlighted the relative importance of direct versus total (direct + indirect) impacts of glacier algae on surface ice albedo and consequently melt (Fig. 6*E*). While congruence was apparent between modeled and measured BBA for lower biomasses of glacier algae (e.g., S9 data, Fig. 6*E*), significant deviation from a 1:1 relationship was apparent at higher algal concentrations (*F*_*1,494*_ = 6.54 × 10^32^, *P* < 0.001), with MODIS-derived BBA consistently lower as compared to modeled BBA. Additional to the direct impacts of glacier algae on surface ice albedo through pigment-mediated energy capture (this study), indirect perturbations of surface ice physics are also likely important (11, 42, 43). Smoother, wetter ice surfaces that develop in concert with increasing glacier algae abundance produce fewer opportunities for high-angle scattering of photons and significant potential for indirect enhancement of the biological albedo reduction effect (11, 42). Our findings confirm the importance of both direct and indirect impacts of glacier algal blooms for processes of GrIS surface darkening. We emphasize that it remains difficult to separate cause and effect since glacier algae may grow preferentially in fast-melting areas where the topographic, hydrologic, and nutrient conditions are favorable and the ice albedo is already low, as well as accelerating melting locally as their biomass accumulates on the ice surface. This represents a melt-accelerating feedback that incorporates both algal growth and physical development of the ice surface and will likely strengthen as bare-ice zones become more expansive and prolonged in warmer climates (7, 11). Future efforts to project ice-sheet mass balance and contributions to sea-level rise must therefore include albedo schemes that account for interlinked biological and glaciological processes operating in the bare-ice zone.

## Conclusions

We have quantified the biological mechanisms underlying darkening and melt of the GrIS associated with blooms of heavily pigmented glacier algae within surface ice. Our data show how significant secondary phenolic pigmentation allows glacier algae to tolerate the irradiance regime apparent within GrIS surface ice, providing significant shading to underlying, low-light–adapted chloroplasts. Photophysiological analyses demonstrated that secondary pigmentation does not represent a rapidly inducible form of photoregulation, with phenolic pigment concentrations remaining relatively stable throughout both short- (24 h) and long-term (2016 ablation season) observations. In this regard, typical NPQ achieved via xanthophyll pigment cycling remains an important means of short-term photoregulation for surface glacier algal communities. By constraining cellular pigment concentrations and biooptical properties of glacier algal phenolic extracts, we were able to demonstrate how secondary pigmentation functions as an effective mechanism to capture UV and lower-wavelength (<465 nm) visible radiation, which is subsequently available to the cell for local meltwater generation. At the meters scale, this mechanism may contribute an additional ∼1.86 cm w.e. meltwater generation per day on the GrIS when algal abundances reach bloom concentrations (∼10^4^ cells⋅mL^−1^). At the regional scale, we show how this mechanism combines with the indirect effects of glacier algae presence within surface ice to drive widespread darkening measured across the southwestern GrIS ablation zone. With larger ablation zones and longer melt seasons expected under a future warming climate, an increase in the magnitude of glacier algal blooms is anticipated given that we show how longer melt duration promotes algal biomass accumulation within GrIS surface ice. Incorporation of biological albedo feedbacks into predictive models of GrIS surface runoff is paramount for rigorous estimates of future ice mass loss and contributions to global sea-level rise.

## Methods

### Data Availability.

All data and associated analysis scripts are available via the Polar Data Centre (44).

### Field Site.

All field sampling, incubations, and photophysiology assessments were performed in situ at our primary ice camp established ∼35 km into the southwestern GrIS (67.04 N, 49.07 W) from 12 July to 20 August 2016. This location lies within the GrIS “dark zone”’ (Fig. 1) and maintained a conspicuous glacier algal bloom throughout the 2016 ablation season (see ref. 23). Glacier algal communities were sampled at regular intervals (every 6 to 10 d) throughout the field season and for specific incubation studies detailed below (*SI Appendix*, Table S1).

### Photophysiology.

An in situ incubation experiment was performed to constrain glacier algae photophysiology under the direct influence of surface ice conditions. Triplicate surface ice areas measuring 20- × 20- × 2-cm depth and containing a conspicuous loading of glacier algal cells (3.6 ± 1.0 × 10^3^ cells⋅mL^−1^) were sampled into sterile Whirl-Pak bags, melted in the dark for 24 h under ambient conditions, and reincubated on the ice sheet surface in triplicate 60-mL biochemical oxygen demand vessels under 100%, 50%, and 0% ambient irradiance for 24 h. This method allowed immediate assessment of photophysiology and pigment profiles on cessation of incubations. Light treatments were achieved using neutral-density filters suspended 10 cm above incubation vessels (50% treatment) or by wrapping vessels in foil (0% treatment). Temperature control was achieved by packing the underside of incubation vessels with snow and ice at regular intervals throughout the incubation period, with no significant difference in incubation temperature apparent between treatments.

After 24-h incubation, measurements of variable chlorophyll fluorescence were performed on 3-mL incubation subsamples with a WaterPAM fluorometer and attached red-light emitter/detector cuvette system (Walz GmBH). Rapid light response curves were performed to constrain glacier algae photophysiology (RLCs; ref. 29), providing information on energy use from limiting through to saturating levels of irradiance (45). All samples were dark-adapted for 20 min prior to RLC assessment, followed by nine 20-s incremental light steps ranging from 0 to 4,000 μmol photons⋅m^−2^⋅s^−1^ with application of a saturating pulse of *ca*. 8,600 μmol photons⋅m^−2^⋅s^−1^ for 600-ms duration at the end of each light step. Maximum quantum efficiency (*F*_*v*_*/F*_*m*_) was calculated from minimum (*F*_*o*_) and maximum (*F*_*m*_) fluorescence yields in the dark-adapted state as *F*_*v*_*/F*_*m*_ = (*F*_*m*_ – *F*_*o*_)/*F*_*m*_. Electron transport through PSII was calculated from PSII quantum efficiency (Y[PSII]) in relative units [rETR = Y(PSII) × incident excitation × 0.5], thereby assuming an equal allocation of excitation between PSI and PSII. To account for light attenuation by glacier algae secondary phenolic pigmentation, incident excitation applied by the fluorometer at λ_660nm_ during RLCs was empirically reduced by the proportion of the total cellular absorption cross-section (discussed in subsequent sections) contributed by phenolic pigmentation at λ_660nm_ (∼94%), and electron transport recalculated from PSII quantum efficiencies as above. Analysis of RLCs (rETR ∼ PAR) followed (29) with iterative curve fitting (R, v.3.6.0) and calculation of the relative maximum electron transport rate (*rETR*_*max*_), theoretical maximum light utilization coefficient (*α*), and light saturation coefficient (*E*_*k*_) following (46). Stern–Volmer NPQ was calculated across RLCs as NPQ = (*F*_*m*_ – *F*_*m*_*′*)/*F*_*m*_, where *F*_*m*_′ represents the maximal fluorescence yield under actinic light.

Simultaneous to photophysiological measurements, a further 5 mL of homogenized sample was fixed with 25% glutaraldehyde at 2% final concentration and transported back to the University of Bristol to assess glacier algal cell abundance (cells⋅mL^−1^) and biovolume (μm^3^⋅cell^−1^) following ref. 23. Total glacier algal biovolume per sample (μm^3^⋅mL^−1^) was calculated as the sum of cell counts multiplied by the average cell biovolume for each species present and converted to units of dry weight biomass (ng DW⋅mL^−1^) after ref. 9. The remaining incubation water (∼50 mL) was filtered across two glass-fiber filters (GF/F; Whatman), which were immediately frozen in a Biotrek-10 cryoshipper (Statebourne) filled with liquid nitrogen. Filters remained under these conditions during transport to the University of Bristol and were stored thereafter at −80 °C prior to pigment analyses (discussed below).

To examine the importance of NPQ for glacier algal photoprotection, inhibitor incubations with dithiorthreitol (DTT) were performed on triplicate melted surface ice samples containing glacier algae. DTT inhibits the xanthophyll epoxidation reactions and thus xanthophyll pigment interconversion, inhibiting the induction of xanthophyll cycle-dependent NPQ as dark-adapted samples transition to illumination (47). Melted samples were treated with DTT at a final concentration of 5 mM for 10 min in the dark, followed by rapid light response curve assessment as above. To serve as comparison, triplicate nontreated control samples were run in tandem.

### Pigmentation.

Regular sampling of glacier algal communities was performed throughout the 2016 ablation season to constrain potential dynamism in pigmentation and to investigate the light absorbance properties of secondary phenolic extracts. A total of 53, 20- × 20- × 2-cm-depth samples of surface ice containing a variable loading of glacier algal cells (187 cells⋅mL^−1^ to 2.1 × 10^4^ cells⋅mL^−1^) were sampled as above throughout this period (*SI Appendix*, Table S1). Following melting in the dark over 24 h and homogenization, ∼100 to 200 mL of each sample was filtered across two glass-fiber filters (GF/F; Whatman), which were immediately frozen and transported as above to the University of Bristol. An additional 15 mL of each sample was fixed at 2% glutaraldehyde final concentration for algal abundance and biovolume determination as above.

For characterization of major chlorophyll and carotenoid pigments, one filter from each pair was freeze-dried for 24 h and extracted in 100% acetone containing vitamin E as internal standard prior to analysis by HPLC. Extracts were analyzed using a modified version of the method of ref. 48, using a c8 column in an Agilent 1100 HPLC equipped with a diode-array detector. Pigments were identified and quantified against analytical standards (DHI and Sigma) using both retention time and spectral analysis. Pigment concentrations were normalized to filtration and extraction volumes. For characterization of water-soluble pigments, the second filter of each sample pair was freeze-dried for 24 h and extracted in Milli-Q water following the method of ref. 26. To remove nonpolar constituents from the raw extract, a phase separation with *n*-hexane was performed. The aqueous phase was then centrifuged and the spectral absorption of the supernant measured with a WPA Light-wave II UV/visible spectrophotometer (Biochrom) from 250 to 750 nm. Concentrations of phenolic extracts were assessed spectrophotometrically using a Gallery Plus Automated Photometric Analyzer (Thermo Fisher Scientific) following Environmental Protection Agency Method 420.1 (49) and examination of extract components performed by HPLC separation and spectral analysis after (26). Cellular pigment quotients were calculated by normalization of pigment concentrations to cell abundance per sample.

Spectral extinction coefficients (L⋅g^−1^⋅cm^−1^⋅nm^−1^, λ_250−750nm_) of phenolic extracts were calculated from the slope of the relationship between absorbance and concentration across all samples as e = A/lc, where A is absorbance, l is path length (cm), and c is concentration (g phenol⋅L^−1^ of melted ice) and transformed after ref. 35 (m^2^⋅mg^−1^). Subsequently, glacier algae single cell absorption cross-sections (a_(λ)_; m^2^⋅cell^−1^) were reconstructed using the in vivo mass-specific absorption coefficients of the major pigment classes (a_i(λ)_, m^2^⋅mg^−1^: phenolics derived here; with values from ref. 34 for chlorophyll *a*, chlorophyll *b*, and carotenoids), multiplied by their cellular contents (C_i_, mg⋅cell^−1^), as a_(λ)_ = Σ a_i(λ)_ × C_i_, representing the virtual area of a completely opaque object blocking the equivalent amount of radiation. For the present study, all carotenoids were considered as photoprotective and the corresponding mass-specific absorption coefficients utilized from ref. 34.

### Energy Budget and IRF.

Single-cell absorption cross-sections were subsequently used to estimate the irradiance absorbed by a representative glacier algal cell from incoming spectral irradiance predicted for our primary ice camp location. Modeled (SPCTRAL2, ref. 50) visible spectral irradiance (W⋅m^−2^⋅nm^−1^; λ_280−750nm_) was downloaded for midday (12 PM) on 26 July 2016 from the PVSystems solar irradiance program (https://www.pvlighthouse.com.au/) after ref. 28. Default settings of the SPCTRAL2 model were accepted and the global (sum of direct and diffuse), perpendicular to the direction of sunlight, spectral irradiance utilized following conversion to units of PAR (μmol photons⋅m^−2^⋅s^−1^). The total amount of irradiance absorbed by a representative glacier algal cell (fmol photons⋅s^−1^⋅nm^−1^) and its constituent major pigment classes (chlorophylls, carotenoids, and phenolics) were calculated by multiplying total or pigment-specific cellular absorption cross-sections (m^2^⋅cell^−1^⋅nm^−1^) by incoming spectral irradiance (μmol photons⋅m^−2^·s^−1^·nm^−1^) and correcting to units of femtomoles (10^9^). Cellular pigment contents were derived from triplicate surface ice samples and associated algal abundance measurements sampled on 26 July 2016. To verify estimates, the total amount of irradiance available to a representative glacier algal cell (fmol photons⋅s^−1^⋅nm^−1^) was calculated by multiplying incoming irradiance (μmol photons⋅m^−2^⋅s^−1^⋅nm^−1^) by half the lateral surface area (expressed in m^2^) of an average-sized *A. nordenskiöldii* cell (cell length = 29.60 ± 7.61 μm, cell width = 12.04 ± 2.06 μm, *n* = 200) and corrected to units of femtomoles (10^9^). These calculations assume a benthic glacier algal cell with the upper portion of the cell absorbing incoming irradiance, consistent with the benthic life history of glacier algae within the ice surface environment and the distribution of pigmentation within glacier algal cells (Fig. 1). Correction for potential pigment packaging effects across wavelengths, that is, greater irradiance absorption than we estimate available to the cell, was achieved by empirically reducing absorbance to the available irradiance at these wavelengths.

Cellular energy budgets were subsequently applied to estimate the IRF posed by a square meter of surface ice containing a low (186 ± 276 cells⋅mL^−1^, *n* = 27), medium (3,711 ± 2,333 cells⋅mL^−1^, *n* = 34), or high (8,989 ± 4,773 cells⋅mL^−1^, *n* = 103) abundance of glacier algae (categories reflecting ref. 23) throughout a complete diel cycle. Spectral irradiance (maintained in units of W⋅m^−2^⋅s^−1^⋅nm^−1^) was downloaded every hour from 12 AM to 11 PM for our primary ice camp location on 26 July 2016 as previously detailed and single-cell energy absorption (expressed as W⋅s^−1^⋅nm^−1^) calculated with correction for pigment packaging effects, minus the contributions of photosynthetic pigments (total chlorophylls). The IRF of a square meter of surface ice (W⋅m^−2^⋅h^−1^⋅nm^−1^) was subsequently calculated by multiplying single-cell energy absorption by low, medium, or high glacier algae abundance (expressed as cells⋅L^−1^), converting from units of volume (L) to surface area (m^2^) using the correction factor of ref. 23, and multiplying by 3,600 s⋅h^−1^. Hourly melt generation (cm w.e.) was determined by integrating IRF across wavelengths (λ_280−750nm_), scaling 1e^4^ to convert square meters to square centimeters and dividing by the latent heat of fusion for melting ice (334 J⋅cm^3^). Daily melt generation (cm w.e.⋅d^−1^) was determined by summing over the complete diel cycle. To estimate uncertainty, calculations were performed for all glacier algal abundances measured per biomass category (total of 164 observations across categories, discussed above), allowing determination of the mean, SD, and SE for estimated melt rates.

### Surface Darkening.

A combination of in situ monitoring, numerical modeling, and remote sensing was used to assess the impact of glacier algae on GrIS surface darkening. Pigment profiles of glacier algae assemblages were monitored at regular intervals across the 2016 ablation season (*SI Appendix*, Table S1) and combined with information on glacier algal biomass modeled at a daily resolution across the southwestern GrIS (K-transect, Fig. 1) to drive the two-stream BioSNICAR-GO radiative transfer model of ref. 11, providing daily estimates of surface ice BBA over the visible spectrum (λ_350–700nm_). Comparisons of glacier algae biomass development and estimated BBA were made to coincident clear-sky MODIS MOD10A1 satellite observations of GrIS surface ice BBA (λ_400–3,000nm_) at 500-m horizontal resolution over our study period (51). An outline of our approach is provided below, with supporting data and associated R scripts provided in *SI Appendix*.

Glacier algal biomass within GrIS surface ice was modeled daily for the entire 2016 ablation season (1 June to 1 September 2016) across the K-transect (Fig. 1) using a reanalysis of the ref. 23 glacier algae bloom development model and information on glacier algae net productivity within surface ice. Previously, ref. 23 modeled glacier algae biomass as a linear function of time since snowline retreat based on field observations across the K-transect using a space-for-time approach. Limitations of this approach include a lack of physical forcing to drive glacier algae growth within surface ice and the resultant unrealistic linear increase in biomass through time. Here, we model daily glacier algal growth across the K-transect based on glacier algal net productivity as a function of the total number of productive hours per day, forced by hourly snowpack thickness (SH), shortwave-down radiation (SWDH), and air temperature (TTH) from reanalysis outputs produced by the regional climate model MARv3.8.1 (52) forced with ERA-Interim at 20-km resolution (*SI Appendix*, Fig. S2).

The environmental prerequisites for algal growth were assessed every hour across our transect to produce daily estimates of the fraction of each 24-h period in which growth occurred. Condition thresholds permissive for growth were chosen through iterative experiments along the K-transect and represent the best compromise to model population sizes representative of measured spatiotemporal dynamics in glacier algal bloom progression (23). Thresholds for growth were thereby set as 1) the ice surface was snow-free (SH < 2 cm), 2) irradiance was sufficient to drive photochemistry (SWDH > 10 W⋅m^−2^), and 3) liquid water was present in surface ice (TTH > 0.5 °C). Optimal daily net growth rates (ng DW⋅mL^−1^⋅d^−1^) were determined from logistic regression of glacier algae net productivity as a function of biomass (both expressed in units of dry weight biomass; see *SI Appendix*, Fig. S3) as assessed during incubation studies by ref. 23 in our primary ice camp location during the 2016 ablation season. The optimal daily glacier algal growth rate was then multiplied by the fraction of daily productive hours to calculate daily net glacier algal growth per location. A daily loss term was incorporated (10% of the population per day) to account for mortality and physical losses of glacier algae from the ice surface (19). For the algal population to experience net growth on any particular day, modeled growth needed to exceed modeled losses.

Measured glacier algal pigment profiles (chlorophyll, carotenoids, and phenolics) and modeled biomass were then input into the BioSNICAR-GO radiative transfer model (11) to calculate daily surface ice albedo integrated over the visible spectrum (λ_350–700nm_), that is, that portion of BBA directly influenced by the light-absorbing effects of glacier algae. Model runs simulated an ice column composed of five layers of ice overlying a flat surface with an albedo of 0.25, representing the solid glacier ice beneath the weathered layer. Each layer was 0.01 m thick with the exception of the upper layer, which was 0.001 m. Ice grains in each layer were assumed to be hexagonal prisms with length and side lengths—in descending order from the surface—of 1,000, 3,000, 5,000, 6,000, and 8,000 µm. Ice densities per layer were 400, 400, 500, 800, and 800 kg⋅m^3^. These values were chosen to reduce the absolute error between simulated spectra with no light-absorbing particles and mean field spectra recorded in situ for clean ice (11). An incoming irradiance spectrum characteristic of midsummer in Greenland was applied (53). All other radiative transfer parameters were set to the default values described in ref. 11. In each daily model run per location, biomass in the upper ice layer was varied to match modeled glacier algal biomass, holding all other variables constant, with glacier algae biomass portioned across three classes of algal cells to reflect cell size distributions measured in situ: 76% of assemblage with cell length = 20 µm and circular end diameter = 12 µm; 15.5% of assemblage with cell length = 60 µm and circular end diameter = 12 µm; 8.5% of assemblage with cell length = 120 µm and circular end diameter = 12 µm. Spectral albedo was integrated over the visible range (λ_350–700nm_; 10-nm resolution) to provide estimated daily BBA per location. Least squares linear regression was applied to compare glacier algal biomass with measured (MODIS) BBA.

## Supplementary Material

Supplementary File
