# Supplementary material for: Algal photophysiology drives darkening and melt of the Greenland Ice Sheet
Source: Proc Natl Acad Sci U S A. 2020 Feb 24;117(11):5694–705. doi: 10.1073/pnas.1918412117 (PMC7084142; doi:10.1073/pnas.1918412117)
Supplement: Supplementary File [file pnas.1918412117.sapp.pdf]

PNAS
www.pnas.org

Supplementary Information for

Algal photophysiology drives darkening and melt of the Greenland Ice Sheet.

Christopher J. Williamson[1*], Joseph Cook[2], Andrew Tedstone[1], Marian Yallop[3], Jenine McCutcheon[4], Ewa Poniecka[5], Douglas Campbell[6], Tristram Irvine-Fynn[2], James McQuaid[4], Martyn Tranter[1], Rupert Perkins[5], Alexandre Anesio[7].

[1]Bristol Glaciology Centre, The University of Bristol, 12 Berkeley Square, Bristol, BS8 1HH, UK; [2]Department of Geography and Earth Science, Aberystwyth University, Penglais, Aberystwyth, Ceredigion, SY23 3FL, UK; [3]School of Biological Sciences, University of Bristol, BS8 1TQ; [4]School of Earth and Environment, University of Leeds, Leeds, LS2 9JT, UK; [5]School of Earth and Ocean Sciences, Cardiff University, Main Building, Park Place, Cardiff, CF10 3AT, UK; [6]Department of Biology, Mount Allison University, Sackville, NB E4L3M7, Canada; [7]Department of Environmental Science, Aarhus University, Frederiksborgvej 399, 4000 Roskilde, Denmark.

Corresponding author
* Christopher J. Williamson.
Email: c.williamson@bristol.ac.uk

This PDF file includes:

Table S1
Figures S1 to S3

www.pnas.org/cgi/doi/10.1073/pnas.1918412117

Table S1. Sampling details over 2016 field campaign. In total, over 200 surface ice samples were collected during the 2016 field expedition, with glacier algal abundance and biovolume assessed for each (see Methods). Average cell dimensions reported in the main manuscript are taken across this complete dataset. Details of sampling pertaining to specific manuscript sections are provided below.

Sampling purpose	Manuscript Section	Date(s) sampled	Replication and sampling comments
Photophysiology incubation study	4.1	Sampled: 19.07.2016 Incubation start: 20.07.2016 Incubation end: 21.07.2016	N = 3 surface ice areas sampled and re-incubated under each of n = 3 light treatments (100%, 50% and 0% ambient irradiance). Total n = 9.
General pigmentation characterization	4.2	Numerous dates (total of 13) ranging 15.07.2016 to 17.08.2016.	N = 53 samples for twinned assessment of chlorophylls, carotenoids and phenolic extracts. Sampling performed across a variety of surface ice habitats (i.e. containing a range of glacier algae abundance) to capture true spatiotemporal dynamics.
NPQ inhibitor incubations	4.3	Sampled: 24.07.2016 Incubated and assessed: 25.07.2016	N = 3 surface ice areas sampled and each assessed ± NPQ inhibitor.
Cellular energy budget	4.4	Sampled: 26.07.2016	N = 3 surface ice areas sampled for pigment and glacier algal abundance quantification.
Temporal dynamism in glacier algae pigmentation	4.5	Sampling dates: 15.07.2016 21.07.2016 30.07.2016 09.08.2016 17.08.2016	N = 43 samples in total, with replication of n = 9, n = 9, n = 5, n = 9, and n = 11, respectively. These samples represent a subset of the n = 53 samples detailed above for general pigmentation characterization.

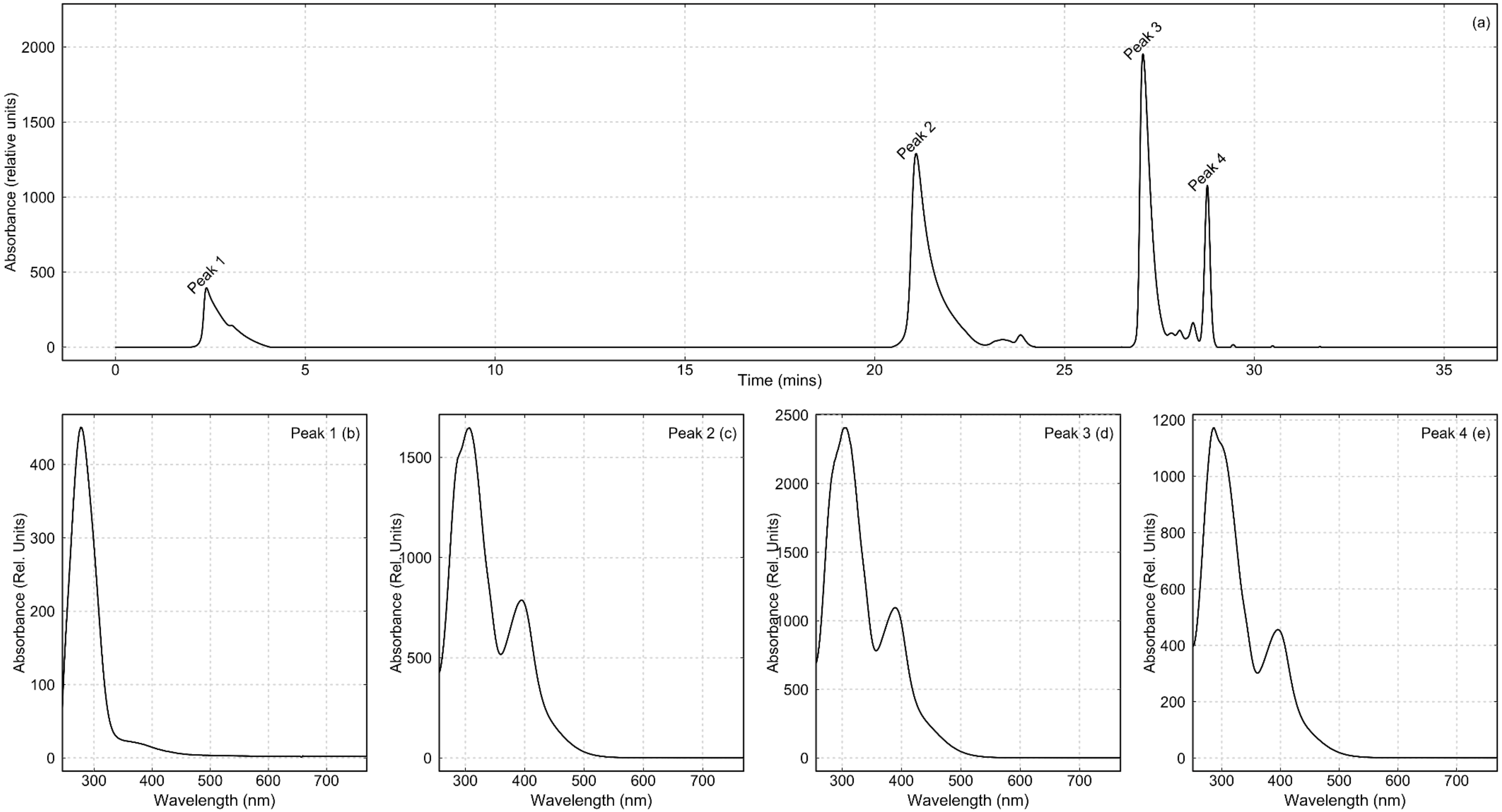

Fig. S1: Representative high-performance liquid chromatography (HPLC) chromatograph (a) and associated absorbance spectra (b – e) of glacier algae water-soluble phenolic extracts, showing the four major compounds present in all samples.

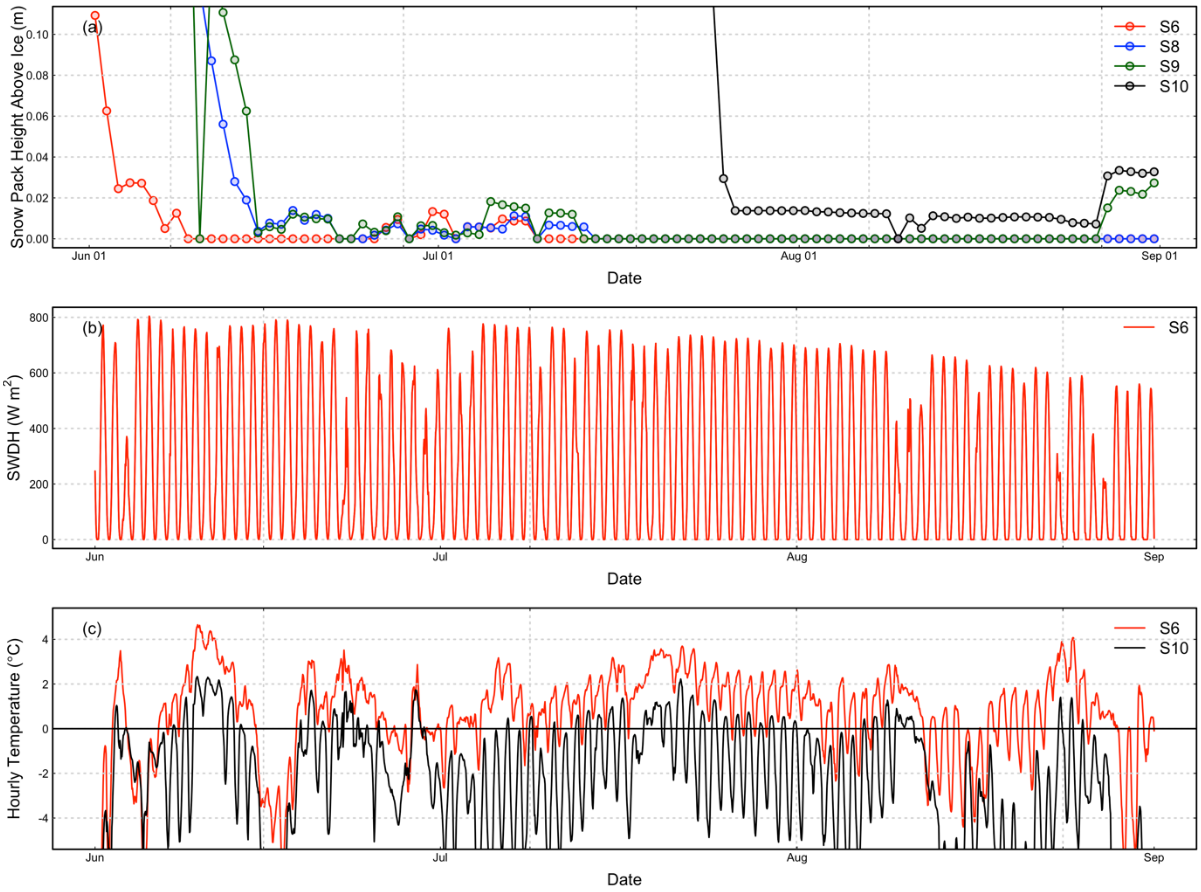

Fig. S2: Representative re-analysis outputs produced by the regional climate model MARv3.8.1 (Fettweis et al. 2017) forced with ERA-Interim at 20 km resolution used to force our glacier algae growth model across the K-transect spanning the southwestern Greenland Ice Sheet (see Methods main manuscript), showing (a) snow pack height above ice across all modelled sites, (b) shortwave downward hourly radiation (SWDH) for site S6 (primary ice camp location) across our model period, and (c) hourly temperature across our model period for our most (S6) and least (S10) marginal sites (See Figure 1 main manuscript).

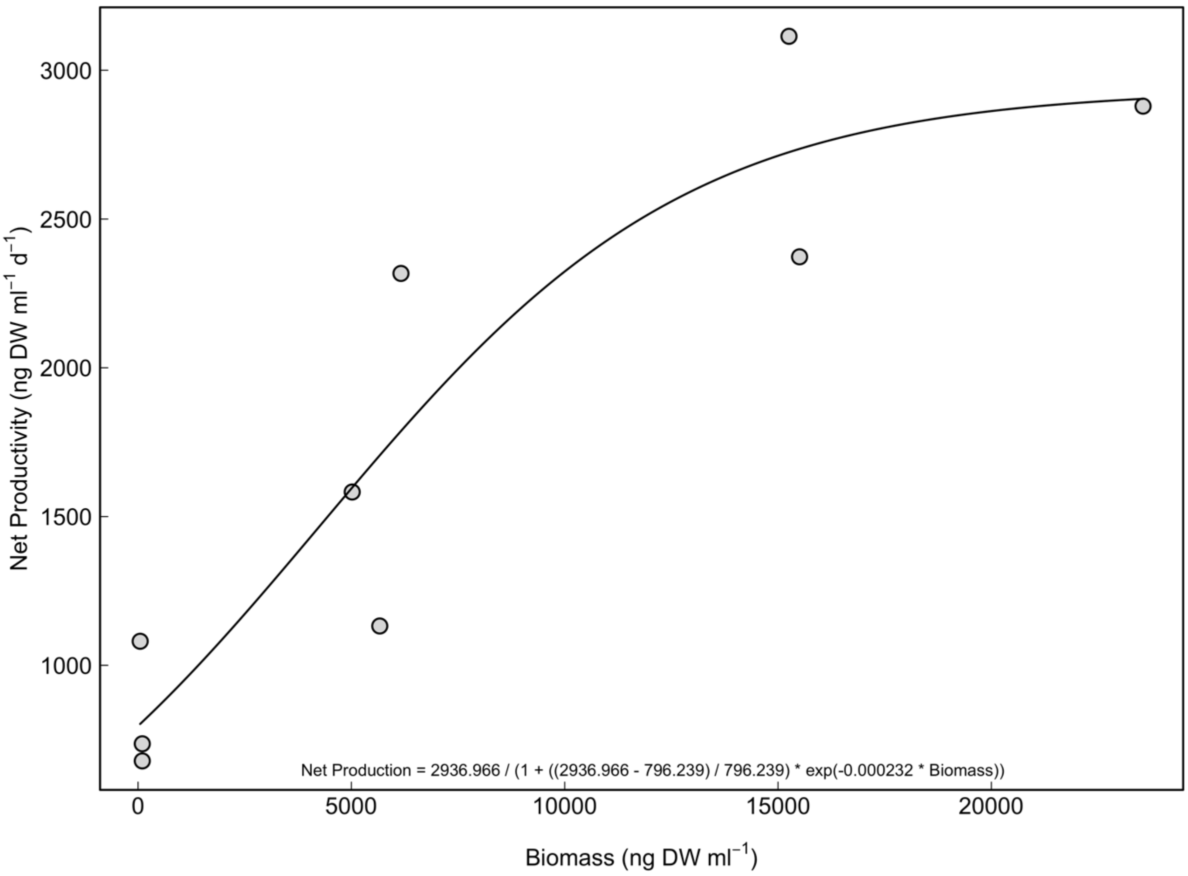

Fig. S3: Glacier algae net productivity (ng DW ml^{-1} d^{-1}) as a function of biomass (ng DW ml^{-1}) modelled using logistic regression as K / (1+ ((K – N0)) / N0) * exp(-r * t)). Data are derived from productivity incubations and associated glacier algal biomass measurements performed by Williamson et al. (2018).

References:

X. Fettweis *et al.*, Reconstructions of the 1900-2015 Greenland ice sheet surface mass balance using the regional climate MAR model. *Cryosphere* **11**, 1015-1033 (2017).

C. J. Williamson *et al.*, Glacier Algae: A Dark Past and a Darker Future. *Front Microbiol* **10**, 524 (2019).
